# Re-examining the iron paradox: the influence of growth medium on regulation and functionality of the tropodithietic acid (TDA) biosynthetic pathway in *Phaeobacter piscinae*

**DOI:** 10.1093/femsec/fiag080

**Published:** 2026-07-17

**Authors:** Lauge Alfastsen, Morgane Mauduit, Michael Scott Cowled, Nikoletta Angela Vig, Robert Friis-Møller, Frederik Valdemar Holck Reimert, Morten Dencker Schostag, Aaron John Christian Andersen, Lone Gram, Sheng-Da Zhang

**Affiliations:** Center for Microbial Secondary Metabolites (CeMiSt), Department of Biotechnology and Biomedicine, Technical University of Denmark, DK-2800 Kgs Lyngby, Denmark; Center for Microbial Secondary Metabolites (CeMiSt), Department of Biotechnology and Biomedicine, Technical University of Denmark, DK-2800 Kgs Lyngby, Denmark; Center for Microbial Secondary Metabolites (CeMiSt), Department of Biotechnology and Biomedicine, Technical University of Denmark, DK-2800 Kgs Lyngby, Denmark; Center for Microbial Secondary Metabolites (CeMiSt), Department of Biotechnology and Biomedicine, Technical University of Denmark, DK-2800 Kgs Lyngby, Denmark; Center for Microbial Secondary Metabolites (CeMiSt), Department of Biotechnology and Biomedicine, Technical University of Denmark, DK-2800 Kgs Lyngby, Denmark; Center for Microbial Secondary Metabolites (CeMiSt), Department of Biotechnology and Biomedicine, Technical University of Denmark, DK-2800 Kgs Lyngby, Denmark; Center for Microbial Secondary Metabolites (CeMiSt), Department of Biotechnology and Biomedicine, Technical University of Denmark, DK-2800 Kgs Lyngby, Denmark; Center for Microbial Secondary Metabolites (CeMiSt), Department of Biotechnology and Biomedicine, Technical University of Denmark, DK-2800 Kgs Lyngby, Denmark; Center for Microbial Secondary Metabolites (CeMiSt), Department of Biotechnology and Biomedicine, Technical University of Denmark, DK-2800 Kgs Lyngby, Denmark; Center for Microbial Secondary Metabolites (CeMiSt), Department of Biotechnology and Biomedicine, Technical University of Denmark, DK-2800 Kgs Lyngby, Denmark

**Keywords:** tropodithietic acid, iron, GFP-reporter fusion, biosynthesis, antimicrobial activity, chemical diversity

## Abstract

Tropodithietic acid (TDA) is a secondary metabolite with antimicrobial and iron-binding properties, produced by several marine *Roseobacter* group bacteria. Although classified as a siderophore, TDA is also produced under iron-replete conditions. To address this paradox, we fused the *tdaCDE* promoter region of *Phaeobacter piscinae* S26 with a promoter-less *gfp* reporter gene to monitor the transcription of TDA biosynthetic genes under different conditions and validated its accuracy using RT-qPCR. In both complex ($\frac{1}{2}$YTIO) and low-complexity (IOCGH) marine media, iron supplementation repressed *tdaCDE* transcription and reduced iron-chelation activity. However, TDA production responded differently depending on medium composition: iron suppressed TDA production in IOCGH but increased it in $\frac{1}{2}$YTIO. Antimicrobial activity of culture supernatants declined in late-stage iron-limited $\frac{1}{2}$YTIO-based cultures, even though *tdaCDE* transcription and iron-chelating activity increased. Acidification restored antimicrobial activity and increased detectable TDA levels, while several non-antimicrobial structural analogues—detected by LC-MS in non-acidified samples—diminished upon acidification, suggesting pH-driven interconversion. These findings reveal that TDA biosynthesis is regulated by iron availability but that the antimicrobial output of the producer depends on medium composition and pH-driven chemical transformations between TDA and its related structural analogues.

## Teaser

Tropodithietic acid biosynthesis in *Phaeobacter piscinae* is influenced by iron and growth medium composition, revealing a versatile secondary metabolite that dynamically balances antimicrobial activity and iron-chelation temporally.

## Introduction

Bacteria from the genus *Phaeobacter* are part of the marine *Roseobacter* group, a globally abundant and metabolically versatile group that occupies different marine habitats ranging from coastal microbial biofilms to open ocean environments (Freese et al. 2017). *Phaeobacter* spp. are often closely associated with marine eukaryotes such as microalgae and bryozoans, where they influence host health, development, and community composition (Seyedsayamdost et al. 2011, Gram 2015, Wang et al. 2016, Sonnenschein et al. 2017, Bech et al. 2023, Bentzon-Tilia et al. 2025). The interactions between *Phaeobacter* and their hosts can range from mutualistic to pathogenic depending on environmental conditions and thus influence phytoplankton bloom dynamics and nutrient cycling in marine ecosystems. Several *Phaeobacter* spp. produce bioactive secondary metabolites that mediate both interactions with the eukaryotic hosts (Wang et al. 2016, 2022) and competitive or cooperative interactions within microbial communities (Sonnenschein et al. 2017, Henriksen et al. 2022, Bech et al. 2023). *Phaeobacter inhibens* and *Phaeobacter piscinae* have been used as model organisms to investigate fundamental ecological processes, including lifestyle transitions between planktonic and biofilm growth, bacterial-algal interactions, and the role of secondary metabolites in structuring microbiomes (Sonnenschein et al. 2017, Henriksen et al. 2022, Bech et al. 2023). A key metabolite underlying many of these ecological functions is tropodithietic acid (TDA) (Rabe et al. 2014, Beyersmann et al. 2017).

TDA shows potent antimicrobial activity against fish pathogenic bacteria and producing bacteria have therefore been explored for probiotic applications in aquaculture (Porsby et al. 2008, Porsby and Gram 2016, Zhao et al. 2016, Beyersmann et al. 2017, Bramucci et al. 2018, Sonnenschein et al. 2021). The antimicrobial activity is hypothesized to occur by disruption of the bacterial proton motive force via an antiporter mechanism (Wilson et al. 2016). TDA can also act as a signaling molecule, mediating quorum sensing (QS) in a manner similar to N-acyl-homoserine lactones (AHLs) (Beyersmann et al. 2017, Henriksen et al. 2022), and as an autoinducer, regulating its own biosynthesis in *P. inhibens* (Geng and Belas 2011) and *Phaeobacter gallaeciensis* (Berger et al. 2011). Given these roles, TDA-producing *Phaeobacter* spp. have been widely studied (Thøgersen et al. 2018, Henriksen et al. 2022, Svendsen et al. 2024, Bentzon-Tilia et al. 2025), but despite significant advances, fundamental questions remain regarding the specific environmental cues and regulatory mechanisms that govern TDA biosynthesis.

Also found to have weak iron-chelation properties (D’Alvise et al. 2016), TDA is hypothesized to serve as a siderophore that enable the producing bacterium to scavenge iron from the natural marine environment (Henriksen et al. 2022). However, unlike traditional siderophores, which are typically only produced under low-iron conditions, TDA is produced in Marine Broth which has a very high (∼0.4 mM) concentration of ferric iron (Zobell 1941, D’Alvise et al. 2016). Moreover, sterile-filtered supernatants from stationary-phase cultures of a TDA-proficient strain of *P. inhibens* only exhibit antimicrobial activity if the cultures grew in iron-rich medium, suggesting that more TDA is produced when iron is abundant, an observation that contradicts the usual behavior of siderophores (Crosa and Walsh 2002, D’Alvise et al. 2016). Under iron-limited conditions in complex nutrient-rich substrates, genes of the TDA biosynthetic pathway in *P. inhibens* are active and lead to production of non-antimicrobial metabolite(s) hypothesized to be TDA structural analogues. Upon acidification, the culture supernatants exhibited antimicrobial activity and TDA was chemically detected (D’Alvise et al. 2016). The chemical identity of the acid-labile, non-antimicrobial analogue(s) of TDA remains unknown.

Further evidence for a link between TDA and iron homeostasis comes from transcriptomic studies of e.g. *Vibrio vulnificus*, which upon exposure to sub-lethal concentrations of pure TDA experienced upregulation of genes associated with siderophore biosynthesis and iron utilization (Dittmann et al. 2019). Likewise, a scarless deletion mutant of *P. piscinae* unable to produce TDA experienced increased transcription and expression of genes related to iron and metal homeostasis in iron-limited medium (Lindqvist et al. 2023). These findings suggest that TDA, directly or indirectly, affects cellular iron availability in both TDA producers and non-producers. The exact mechanism by which TDA binds and imports iron remains unknown. However, the observation that TDA and related metabolites are produced under both iron-limited and iron-replete conditions, yet display variable antimicrobial activity (D’Alvise et al. 2016), suggests a complex relationship between iron availability, TDA biosynthesis and functional output. Together, this gives rise to an apparent paradox: although TDA exhibits iron-chelating properties and influences iron homeostasis, its production, as reflected by antimicrobial activity, appears to be enhanced under iron-replete conditions.

Here, we address the contradiction between TDA as an iron-chelating molecule and its production under iron rich conditions. Our purpose was to investigate if the apparent higher production and antimicrobial activity of TDA in the presence of iron was a direct function of higher expression of TDA biosynthetic genes. To address this, a plasmid-based fluorescent reporter was built and validated by RT-qPCR as an accurate method to estimate the transcription of the core TDA biosynthetic gene operon *tdaCDE*, which allowed us to determine the temporal dynamics of TDA biosynthesis under different growth conditions, focusing on the influence of iron. This was coupled, when possible, to bioassays and chemical detection of TDA, along with its structural analogues, to describe the production and activity of this multifaceted secondary metabolite.

## Materials and methods

### Strains, media, and growth conditions

All strains used in this study are listed in Table 1. Marine bacteria, i.e. *P. piscinae* S26 (Sonnenschein et al. 2017b), its TDA-deficient mutant S26 Δ*tdaB* (Lindqvist et al. 2023), *Pseudoalteromonas piscicida* S2040 (Sonnenschein et al. 2017a) and *Vibrio anguillarum* 90–11–286 (Pedersen et al. 1997), were grown non-shaken at 25°C in different media depending on the purpose. Marine Broth 2216 (MB, BD 279110, BD Difco) and Marine Agar 2216 (MA, BD 212185, BD Difco) were routinely used to maintain the strains and grow pre-cultures. An MB-equivalent medium with 0.2%(w/v) yeast extract, 0.125%(w/v) tryptone, and 2%(w/v) Instant Ocean Sea Salts (IO, Prod. SS15-10, Aquarium Systems Inc.) ($\frac{1}{2}$YTIO) was used to enable cultivation with and without iron [inspired by $\frac{1}{2}$YTSS (González et al. 1996)]. A simpler marine medium-based on the Instant Ocean salts [IOCGH: 3%(w/v) IO, 0.3%(w/v) casamino acid, 0.2%(w/v) glucose and 0.3% HEPES (Lindqvist et al. 2023)] was used to monitor TDA biosynthetic gene expression as influenced by iron and other factors. When needed, the base media were supplemented with FeCl_3_ [Iron(III) chloride hexahydrate, 236 489, Sigma–Aldrich)] at a final concentration of 0.5 mM, or FeSO_4_ [Iron(II) sulfate heptahydrate, 215 422, Merck], ZnCl_2_ (208 086, Sigma–Aldrich), or NiCl_2_ [Nickel(II) sulfate hexahydrate, 223 387, Sigma–Aldrich] in a range of concentrations. *Phaeobacter* strains carrying pBBR1-MCS derived plasmids were cultured in MB supplemented with 200 μg/ml kanamycin to maintain the plasmid in pre-cultures. The *Escherichia coli* strain Top 10 was grown in LB broth medium (BD 244620, BD Difco) or on LB agar (LA) plates (BD 244520, BD Difco) at 37°C. The *E. coli* strain WM3064 was cultured in LB or LA supplied with 0.03 mM diammonium phosphate (DAP) at 37°C. All *E. coli* strains carrying pBBR1-MCS derived plasmids were cultured in media with 50 μg/ml kanamycin.

**Table 1:** Strains used in this study.

Strains	Genotype/feature	Reference or source
***Escherichia coli***		
Top 10	F^−^*mcrA* Δ(*mrr*-*hsdRMS*-*mcrBC*) *φ80 lacZ* Δ*M15* Δ *lacX74 recA1 ara*Δ*139*Δ(*ara*-*leu*)7697 *galU galK rpsL* (StrR) *endA1 nupG*	Invitrogen^TM^
WM3064	*thrB1004 pro thi rpsL hsdS lacZ*Δ*M15 RP4-1360* Δ*(araBAD)567* Δ*dapA1341::[erm pir]*	William Metcalf at UIUC
***Phaeobacter piscinae***		
S26 WT	Wild type	(Grotkjær et al. 2016)
S26 Δ*tdaB*	TDA deficient mutant, with scarless deletion of *tdaB* gene	(Lindqvist et al. 2023)
S26-NC	S26 carrying the empty plasmid pBBR1-MSC2	This study
S26-P*_tdaCDE_*-GFP	S26 carrying the reporter plasmid pBBR1-MSC1-P*_tdaCDE_*-GFP	This study
***Pseudoaltermonas piscicida***		
S2040	Wild type, producer of several siderophores	(Gram et al. 2010, Sonnenschein et al. 2017)
***Vibrio anguillarum***		
90–11–286	Wild type, isolated from diseased rainbow trout	(Pedersen et al. 1997)

### Construction of fluorescent reporter strain

All plasmids and primers used in this work are listed in Tables 2 and 3, respectively. To construct the reporter plasmid, the promoter region and 186 bp-5′ of *tdaC* gene were amplified by primer pair P*tdaB*’C-F and P*tdaC*-gfp-R and fused to the 5′ of a promoter-less GFPmut3* gene amplified by primer pair GFPmut3-F/-R. The fused segment was subcloned to pJet1.2 vector *via* the Rapid DNA ligation Kit (Thermo Scientific, cat. K1423) and finally cloned onto vector pBBR1-MCS2 via cloning site XhoI and XmaI using the fast cloning kit from New England BioLab (M2200, Bionordika, DK) according to the manufacturer’s protocol. Recombined plasmids pBBR1-MCS2-P*_tdaC_*GFPmut3* were verified by sequencing by Macrogen Europe (NL) and electroporated into *E. coli* WM3064. Conjugation (Lindqvist et al. 2023) was carried out to deliver pBBR1-MCS2-P*_tdaC_*GFPmut3* into *P. piscinae* S26 to generate the GFP-tagged strain denoted S26-P*_tdaCDE_*-GFP (Table 1). The empty vector pBBR1-MCS2 was conjugated into S26 to generate strain S26-NC (Table 1), which was used as a negative control.

**Table 2:** Plasmids used in this study.

Plasmid	Features	Reference or source
pJET1.2	pMB1 origin of replication, Amp^r^, P_lacUV5_, *eco47IR*, T7 promoter	ThermoFisher Scientific
pJET1.2-P*_tdaCDE_-*GFPmut3*	pJET1.2 backbone carrying the promoter region of *tdaCDE* of S26 fused to a promoterless GFPmut3* gene	This study
pBBR1-MCS2	pBBR1 origin of replication, Kan^r^, P_lac_, *lacZα*	(Obranić et al. 2013)
pBBR1-MCS2-P*_tdaCDE_*-GFPmut3*	pBBR1-MCS2 backbone carrying the promoter region of *tdaCDE* of S26 fused to a promoterless GFPmut3* gene	This study

**Table 3:** Primers used in this study.

Primer	Sequence (5´- 3´)	Application
Check-Pgfp-F	ATGCGTAAAGGAGAAGAACTTTTC	Verification of the GFPmut3* gene
Check-Pgfp-R	TTATTTGTATAGTTCATCCATGC	
PtdaB’C-F	AAAACTCGAGCTGGTTTGAGCGCTGTCT	PCR-amplification of the promoter region of *tdaCDE* operon from *Phaeobacter piscinae* S26 for cloning
PtdaC-gfp-R	TTCTCCTTTACGCATGGCCTGTTCCATCGTCC	
GFPmut3-F	ACGATGGAACAGGCCATGCGTAAAGGAGAAGAACTTTTC	PCR- amplification of the promoterless GFPmut3* gene for cloning
GFPmut3-R	ATCCCGGGTTATTTGTATAGTTCATCCATGC	
tdaC-F	TTTTGGTTGGCGTGGTGG	RT-qPCR analysis of *tdaC* transcription of *Phaeobacter piscinae* S26 strains
tdaC-R	ATCGGGAGCAACTTTTCAG	
rpoB-F	AAACGGGCATCCAGAGCA	RT-qPCR analysis of *rpoB* transcription of *Phaeobacter piscinae* S26 strains
rpoB-R	AGATCGAGCGTGAAGAAGT	

### Fluorescence microscopy

The reporter strain S26-P*_tdaCDE_*-GFP and the control strain S26-NC (Table 1) were streaked on MA plates or grown in MB without kanamycin for at least 48 h. Fluorescence of the colonies was detected under a Zeiss microscope with modes of bright field or green fluorescence (ex. 485 nm/20 nm; em. 520 nm/20 nm). Images were acquired with the ZEN software. The fluorescence of individual cells in suspension was observed and photographed using a Nikon inverted microscope as described in Bech *et al*. (Bech et al. 2023). The exposure time was the same for GFP-tagged and control cultures. Images were processed in ImageJ (Schneider et al. 2012) with the same parameter adjustments.

### Growth and fluorescence kinetics assay

Three individual colonies of the reporter strain S26-P*_tdaCDE_*-GFP and the control strain S26-NC were used to establish biological triplicate pre-cultures in MB. After 24 h of growth, the pre-cultures were diluted to 10^3^–10^4^ CFU/ml in the respective growth media. Cultures were distributed as 100-µl aliquots into the wells of a 96-well Nunc^TM^ MicroWell Optical-Bottom Plates (Thermo Fisher Scientific, cat no. 165 305) and incubated for 72 h with optical density at 600 nm (OD_600_) and GFP (ex. 480/15 nm; em. 517/15 nm) relative fluorescence intensity (RFI; arbitrary units) recorded every 20 min in an Agilent BioTek Synergy H1 multimode reader (AH diagnostics).

The data processing was performed using OriginLab (Version 2025) and R (R Core Team (2022). OD_600_ and RFI data points from each microplate-well were background-corrected by subtracting the corresponding media negative control reads. The background corrections were applied only to OD_600_ and RFI values above 0 to avoid magnification of negative value outliers. The OD_600_ values and RFI values normalized to optical density (RFI/OD_600_) were plotted in OriginLab Version 2025. The OD_600_ vs time graphs are plotted using locally estimated scatterplot smoothing (LOESS), with a very minor smoothing span = 0.2. This was done to visualize the growth phases more defined along with a rolling standard deviation.

### RNA extractions and RT-qPCR analysis

Pre-cultures of the reporter strain S26-P*_tdaCDE_*-GFP and the wild-type (S26 WT) were set up in biological triplicates in MB as described previously. After 24 h of incubation, pre-cultures were diluted to 10^4^–10^5^ CFU/ml in 200 ml IOCGH medium in Erlenmeyer flasks and allowed to incubate non-shaken at 25°C for 60 h. Culture samples were taken every 12 h to monitor cell density [measured as colony forming units (CFU) per ml], green fluorescence (measured with same settings as above) and mRNA levels.

For RNA extractions, 0.5 ml aliquots of bacterial cultures were mixed with 1 ml RNAprotect Bacteria Reagent (QIAGEN), followed by centrifugation at 9000 × g for 10 min. Cell pellets were flash-frozen in liquid nitrogen and stored for up to one week at −80°C. RNeasy Protect Bacteria Mini Kit (QIAGEN) was used to extract total RNA from samples following the manufacturer’s instructions. Residual DNA was removed from extracts using the Turbo DNA-free kit (Invitrogen). The template from RNA extraction was 20 µl, mixed with 2 µl 10X TURBO DNase buffer and 2 µl TURBO DNase Enzyme and 5 µl DNase Inactivation Reagent. From the purified RNA, SuperScript IV Reverse Transcriptase (Invitrogen) was used to synthesize the cDNA to be used as template in the quantitative PCR (qPCR). Primers amplifying *tdaC* and *rpoB* genes were designed with CLC Genomics Workbench. The qPCR assay was performed with 10 µl Luna SYBR Green, 0.5 µl (0.25 µM, final concentration) forward and reverse primer (Table 3) of the *tdaC* or *rpoB* gene, 8 µl H_2_O and 1 µl DNA template sample. The PCR reaction was performed with a starting temperature of 95°C, followed by 40 cycles of 95°C for 15 s and 60°C for 30 s.

The data acquired from RT-qPCR was analyzed with Bio-Rad CFX Maestro 2.3 (version: 5.3.022.1030). Melting curve analysis confirmed the specificity of PCR amplification using positive controls (DNA template) and negative controls (DNase-treated RNA). Relative standard curves were made by dilution series of samples with known cell densities (CFU/ml) from the last time point in the corresponding PCR plate, one S26 WT and one S26-P*_tdaCDE_*-GFP. Starting quantities (SQ/ml) of cDNA were calculated by CFX Meastro (v2.3) based on linear regression of cycle threshold values against the log-transformed initial template quantity of the standards. CFU/ml, GFP RFI and mRNA SQ/ml measurements were plotted in R using the ggplot2 package (ggplot2: Elegant Graphics for Data Analysis, https://ggplot2.tidyverse.org).

### Liquid CAS assay

Iron-chelating activity of culture supernatants was quantified using the liquid chrome azurol S (CAS) assay (Himpsl and Mobley 2019). Briefly, non-shaken cultures of *P. piscinae* S26 (Table 1) were grown for 72 h in IOCGH medium with and without 0.5 mM ferric chloride or for 72 h in $\frac{1}{2}$YTIO medium without added iron, with culture aliquots taken every 24 h. When needed, cultures of the TDA-deficient mutant S26 Δ*tdaB* (Table 1) grown under identical conditions were included as negative control. All *c*ultures were set up in biological triplicates. The potent siderophore-producer *Pseudoalteromonas piscicida* S2040 (Table 1) grown in either IOCGH or $\frac{1}{2}$YTIO medium was used as a positive siderophore control. Sterile-filtered culture supernatants were prepared by centrifuging culture aliquots at 9000 × g for 5 min and filtering the supernatants through 0.2 µm Minisart ® NML Syringe Filters (Cat#16 534 K, SARTORIUS). For the CAS assay, 100 µl of sterile-filtered culture supernatant or 100 µl of the corresponding sterile growth medium was mixed 1:1 with CAS assay solution in a 96-well microtiter plate. Mixtures incubated for 20 min at room temperature, after which absorbance at 630 nm was measured in a BioTek Synergy H1 plate reader. The relative iron-chelating activity is calculated as A_s_/A_r_, where A_s_ represents the absorbance of CAS solution mixed with supernatant and A_r_ represents the absorbance of CAS solution mixed with sterile medium. Lower A_s_/A_r_ values indicate higher iron-chelating activity (Himpsl and Mobley 2019). Visual inspection of mixtures was done to confirm the color change from blue to orange or light gray in mixtures.

### Well-diffusion inhibition assay

Samples for the inhibition assays and the UHPLC-MS analyses (described in later section) were obtained from non-shaken *P. piscinae* S26 cultures grown in 40 ml medium (in biological triplicates) with 5-ml aliquots collected at 24, 48, and 72 h. pH was measured of every collected culture aliquot and of a non-inoculated medium control using pH indicator strips from Cytiva (pH 4.5–10, Cat # 2614–991). For the inhibition assay, sterile-filtered culture supernatants prepared as previously described were split into two 700-µl aliquots, one acidified to pH 1.5–2.0 by addition of 70 µl 0.3 M hydrochloric acid and the other non-acidified by addition of 70 µl distilled water. The ability of the acidified and non-acidified supernatants to inhibit the growth of *V. anguillarum* 90–11–286 (Table 3) was tested by well-diffusion, using a protocol adapted from (D’Alvise et al. 2013). Pre-cultures of *V. anguillarum* 90–11–286 were grown for 24 h in 5 ml MB with agitation at 200 RPM. The assay plates were prepared by adding 50 µl of the *V. anguillarum* pre-culture and 1 ml of a 20% (w/v) glucose solution into 50 ml molten IOC agar (per liter: 10 g bacteriological grade agar, 30 g Instant Ocean sea salts, 3.3 g casamino acids, dissolved in distilled water) at 45°C, mixing, and gently pouring into 120 × 120 × 17 mm square-shaped petri dishes (Cat # 11798563, Fisher Scientific). Once solidified, 7 mm wells were punched into the solid media aseptically. Fifty microliters of sterile-filtered culture supernatants were added to the wells of the *Vibrio*-embedded agar plates. As a negative control, a non-inoculated medium was processed and applied by the same protocol. As positive control, a sterile-filtered supernatant prepared from a 48 h non-shaken culture of *P. piscinae* S26 grown in MB was also included. Plates were incubated at 25°C for 2 days. The diameter of the inhibition zone (including the well diameter) was measured. Lower limit of detection (LOD) of this assay was 7 mm.

### Sample preparation for UHPLC-MS

Culture aliquots of *P. piscinae* S26 collected at 24, 48, and 72 h described in the previous section were prepared for UPHLC-MS analysis by liquid-liquid extractions. Briefly, extractions were performed (in technical triplicates) by mixing 0.9 ml of culture aliquot with an equal volume of HPLC-grade ethyl acetate (EtOAc, VWR Chemicals) containing 1% formic acid, incubating at room temperature for 30 min with whirl-mixing every 10 min, centrifuging at 10 000 × g for 5 min, and collecting 0.7 ml of the upper (organic) phase. Two sequential extractions were performed for each technical replicate, with organic phases combined subsequently. The collected organic phases were dried under nitrogen for 1.5 h and redissolved in 100 µl 85% (v/v) acetonitrile-H_2_O (LC-MS grade, VWR Chemicals).

### Sample preparation for UHPLC-MS/MS

Non-shaken cultures of *P. piscinae* S26 were grown for 72 h in $\frac{1}{2}$YTIO medium, and sterile-filtered culture supernatants, acidified and non-acidified, were prepared as previously described. To avoid further acidification during sample preparation prior to UHPLC-MS/MS analysis, 2.4 ml of supernatants were frozen at −80°C and dried *in vacuo* overnight, redissolved in 200 µl methanol (LC-MS grade), centrifuged at 10 000 × g for 2 min, sonicated for 3 min, and centrifuged again. The methanol-phases were collected and analyzed immediately (Day 0 detection) by UHPLC-MS/MS. Samples were reanalyzed after <24 h of storage at 4°C, followed by an 8-day storage at −20°C (Day 9 detection).

### UHPLC-MS and UHPLC-MS/MS data acquisition and analysis

Liquid chromatography–high-resolution mass spectrometry (LC-HRMS) was performed at the DTU Metabolomics Core Facility using an Agilent 1290 Infinity II UHPLC system (Agilent Technologies, Santa Clara, CA, USA) coupled to a Bruker TIMS TOF Flex mass spectrometer equipped with an electrospray ionization (ESI) source. Extracts (2 µl injection volume) were separated on an Agilent Poroshell 120 Phenyl-Hexyl column (2.1 × 150 mm, 1.9 µm) maintained at 40°C. Mobile phases consisted of water (A) and acetonitrile (B), both containing 20 mM formic acid. Chromatographic separation was achieved using a linear gradient from 10% to 100% B over 10 min, followed by an isocratic hold at 100% B for 2 min, before returning to starting conditions. The flow rate was 0.35 ml/min. Ultraviolet (UV)−visible spectra were recorded from 190 to 640 nm, and UV absorbance at 385 nm was used for semi-quantitative analysis of TDA based on integrated peak areas of the chromatographic signal. Source parameters were set as follows: drying gas temperature 220°C, drying gas flow 10 l/min, capillary voltage 3500 V, and end plate offset 500 V. Data were acquired as centroid spectra across a *m/z* range of 100–1400. For targeted analysis of TDA (UHPLC-MS) spectra were acquired in positive ionization mode at a rate of 4 spectra/s without DDA fragmentation. For targeted analysis of analogues (UHPLC-MS/MS) spectra were acquired in negative ionization mode at a rate of 10 spectra/s, with DDA fragmentation at three collision energies (15, 25, and 40 CeV).

Analysis of LC-MS data was performed with the Compass DataAnalysis software suite from Bruker Daltonik GmbH (version 5.0). The presence of TDA in samples was confirmed by comparison to the retention time, isotopic pattern and accurate mass of an authentic standard run in parallel. From the UHPLC-MS data (positive ionization), the relative abundance of TDA in culture extracts was calculated as the sum of the peak areas of the extracted ion chromatograms (EICs) of the two dominant adducts of TDA: the proton adduct ([TDA+H]^+^, *m/z* 212.9674 ± 0.002) and the sodium adduct ([TDA+Na]^+^, *m/z* 234.9494 ± 0.002). From the UHPLC-MS/MS data (negative ionization), the EIC of the deprotonated molecular ion of TDA ([TDA-H]^−^, *m/z* 210.9528 ± 0.002) was used and analogues of TDA were identified based on the presence of the shared fragment ion (*m/z* 106.9961 ± 0.002) in their spectra, and further validated based on the isotopic pattern of the precursor ion.

### Statistics

Unless stated otherwise, all quantitative experiments were performed with biological triplicates, i.e. three individual colonies used to set up three separate pre-cultures. Pairwise statistical comparisons between different time points, media, and treatment conditions were performed using two-sided Welch’s *t*-test. Resulting *P*-values were adjusted for multiple testing using the Benjamini–Hochberg false discovery rate (FDR). A pearson correlation coefficient was calculated to assess the linear relationships between *tdaC* expression, as quantified by RT-qPCR, and *tdaCDE* promoter activity, as quantified by GFP RFI. Statistical analyses were conducted in R using base functions and packages.

## Results

### Plasmid-based fluorescent reporter strain monitors the transcription of *tdaC*.

**Figure 1:**
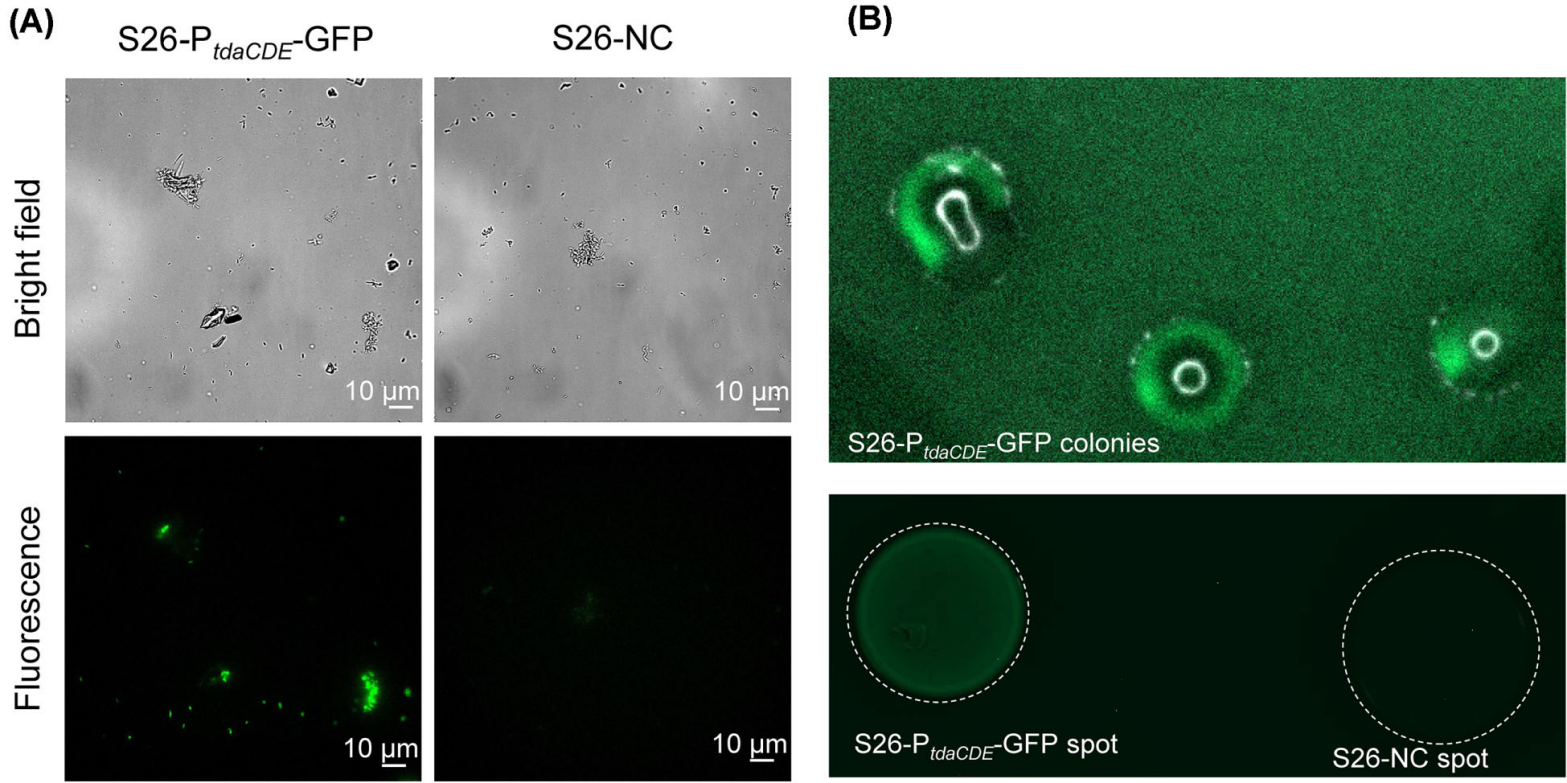
Detection of the GFP fluorescence in *P. piscinae* strains. Bright field (A, top) and inverted epifluorescence (A, bottom) microscopy images of *P. piscinae* S26 grown in marine broth (MB), either carrying the reporter plasmid pBBR1-MCS2-P_tdaCDE_-GFP (S26-P*_tdaCDE_*-GFP) or the empty plasmid pBBR1-MCS2 (S26-NC). GFP fluorescence (B) on marine agar (MA) plates. The top panel shows colonies of the reporter strain and the bottom panel shows spotted cultures of the reporter strain and the non-fluorescent control after 24 h of growth.

We constructed a plasmid-based reporter by fusing a green fluorescent protein (GFP) gene with the promoter region of the TDA biosynthetic gene operon *tdaCDE* from *P. piscinae* S26, a potent TDA-producing strain isolated from an algal microbiome (Grotkjær et al. 2016). The *P. piscinae* S26 reporter strain (denoted S26-P*_tdaCDE_*-GFP, Table 1) gave detectable GFP emission when grown in Marine Broth (MB) and the signal exceeded the autofluorescence of the control strain (S26-NC) carrying the empty plasmid, both at single-cell level from liquid cultures (Fig. 1A) and at colony level from solid media (Fig. 1B). The reporter plasmid harbors a kanamycin resistance marker, but growth and fluorescence measurements from cultures with and without the antibiotic demonstrated similar average fluorescence levels across growth phases and all subsequent experiments were therefore conducted without kanamycin.

In general, the reporter plasmid did not seem to impose any fitness burden, as the growth of S26-P*_tdaCDE_*-GFP was comparable to that of the wild type S26 (no significant difference between growth rates, Welch’s *t*-test, *P* = 0.80) (Figure S1A–B) and expression of the housekeeping gene *rpoB* developed at similar levels in cultures of both strains (Figure S1C). The temporal expression pattern of *tdaC*, measured by RT-qPCR aligned closely with GFP signal accumulation in the reporter strain (Figure S1D): Across all time points, GFP relative fluorescence intensities (RFI) and *tdaC* transcript levels showed good linear correlation (Pearson, r = 0.84), demonstrating that the S26-P*_tdaCDE_*-GFP reporter strain closely monitors native promoter activity.

### Presence of iron during cultivation represses TDA biosynthetic gene transcription and iron chelation activity

Investigation of iron influence on TDA biosynthesis is not feasible with MB due to the intrinsic iron content of this medium (Zobell 1941). Instead, two different base media were used: The $\frac{1}{2}$YTIO medium, a yeast extract tryptone recipe mimicking the $\frac{1}{2}$YTSS medium widely used by marine microbiologists (González et al. 1996, Moran et al. 2007, D’Alvise et al. 2016), was chosen for having similar nutrient richness as MB. The IOCGH medium, containing ocean salts, casamino acids, glucose and HEPES, was chosen as a low-complexity alternative (D’Alvise et al. 2010, Lindqvist et al. 2023).

**Figure 2:**
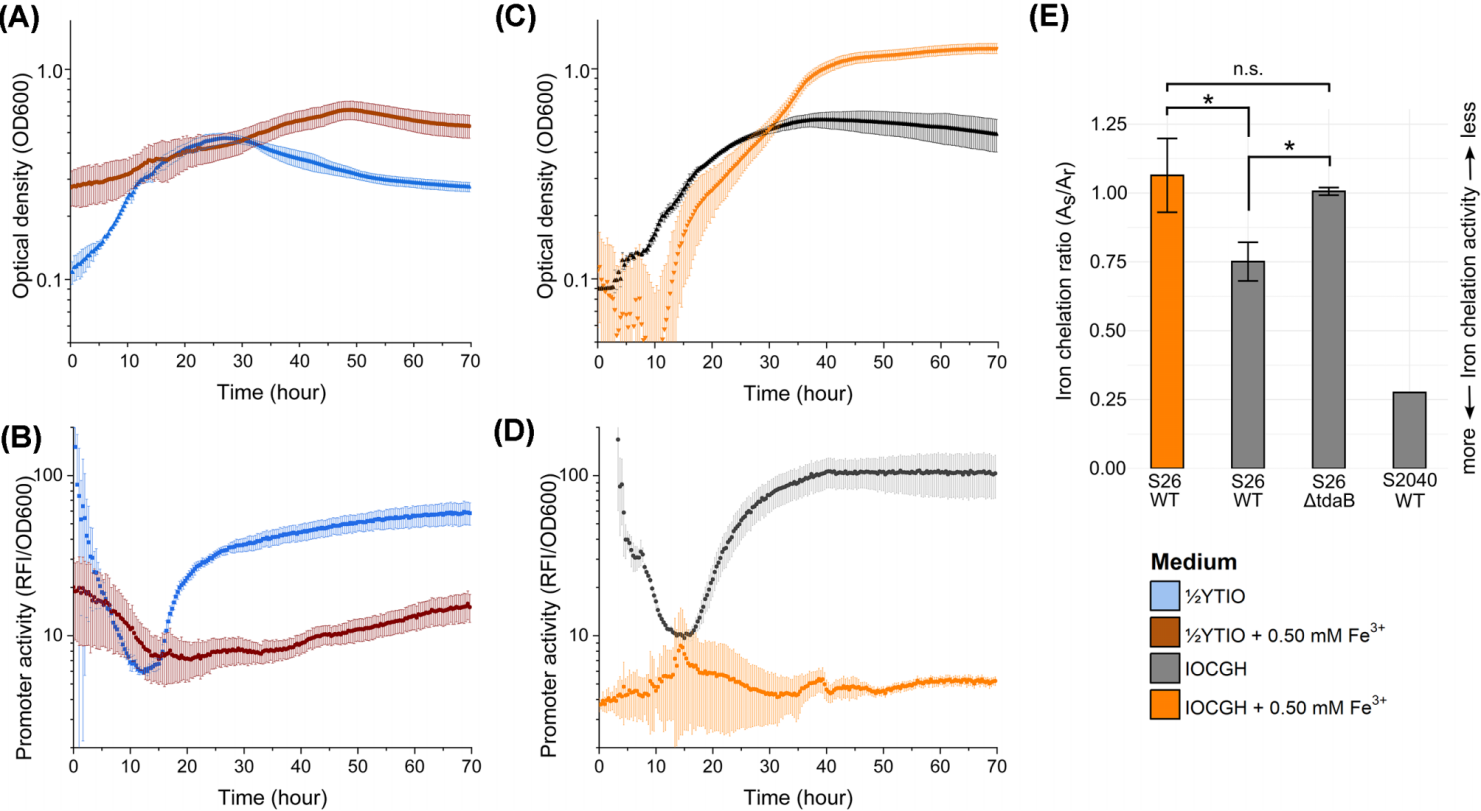
Iron availability modulates tropodithietic acid (TDA) biosynthesis andiron-chelating activity in a manner expected for a siderophore metabolite. Three individual colonies (constituting biological triplicates) of the reporter strain *P. piscinae* S26-P*_tdaCDE_*-GFP were cultured overnight in Marine broth and followingly used to start assay cultures in $\frac{1}{2}$YTIO medium (A, B) and IOCGH medium (C, D), with or without 0.50 mM Fe³⁺. Optical density (OD₆₀₀) and GFP relative fluorescence intensity (RFI; arbitrary units) were measured every 20 min over 72 h. Panels A and C show growth curves, while B and D display activity of the *tdaCDE* promoter, calculated as RFI normalized by OD₆₀₀. Lines represent the mean of the biological triplicates; shaded regions show standard deviations. Note that sterile media with iron has a higher basal optical density than sterile medium without iron. (E) Iron-chelating activity of 72-h sterile-filtered culture supernatants was assessed using the liquid Chrome Azurol S (CAS) assay. Results are shown as the ratio of absorbance of CAS solution mixed with culture supernatant relative to a sterile medium control (A_s_/A_r_), where lower values indicate stronger iron chelation. Supernatants of wild-type S26 grown with or without iron and the TDA-deficient mutant S26Δ*tdaB* grown without iron were compared to supernatants of the potent siderophore-producer *Pseudoalteromonas piscicida* S2040. Bars represent the mean of biological triplicates ± standard deviation. Statistical comparisons were made using two-sided Welch’s *t*-tests with Benjamini–Hochberg correction. Significance thresholds: *P* < 0.05 (*); n.s., not significant.

Supplementation of $\frac{1}{2}$YTIO medium and IOCGH medium with 0.50 mM ferric iron (Fe^3+^) increased the maximum cell densities of *P. piscinae* S26 cultures (Fig. 2A and **C**). The *tdaCDE* promoter activity, measured as GFP RFI normalized to optical density (RFI/OD_600_), decreased markedly under iron-supplemented conditions in both media but to different extents. In $\frac{1}{2}$YTIO-based cultures, normalized promoter activity in iron-limited cultures had an approximately three-fold higher maximum than iron-supplemented cultures (Fig. 2B), whereas the RFI/OD_600_ maximum in IOCGH-based cultures was roughly ten-fold higher in iron-limited conditions than in iron-supplemented conditions (Fig. 2D). Supplementation with ferrous iron (Fe^2+^) also caused repression of *tdaCDE* promoter activity (Figure S2A), while supplementation with other metal ions, including zinc and nickel, did not cause any significant changes (Figure S2B-C). Thus, *tdaCDE* transcription is specifically repressed by iron and maximized under iron limitation, similar to canonical siderophore biosynthesis.

We further tested whether actual iron-chelation activity was restricted to cultures grown without iron. Sterile-filtered supernatants from cultures of *P. piscinae* S26 WT grown in IOCGH medium without iron supplementation did have measurable iron-chelation activity when analyzed by the liquid CAS assay (Fig. 2E), albeit not as strong as the iron-chelation activity of sterile-filtered culture supernatant from the potent siderophore producer *Pseudoalteromonas piscicida* S2040. This activity was abolished in cultures of S26 WT supplemented with iron and in cultures without added iron of *P. piscinae* S26ΔtdaB (Table 1), a scarless deletion mutant incapable of producing TDA (Lindqvist et al. 2023), suggesting that the observed iron chelation depends on an intact and actively transcribed TDA biosynthetic pathway.

### Iron has opposite effects on TDA production depending on the growth medium.

Since the presence of iron caused both lower transcription of the *tdaCDE* operon and lower iron-chelation activity by S26 WT cultures, we investigated whether this siderophore-like response is also reflected by lower levels of produced TDA in cultures supplemented with iron. We measured TDA production in cultures over the course of three days by UHPLC-MS of culture extracts, a semi-quantitative approach used previously by others to monitor the production of marine microbial secondary metabolites (Bose et al. 2015, Droumpali et al. 2023).

**Figure 3:**
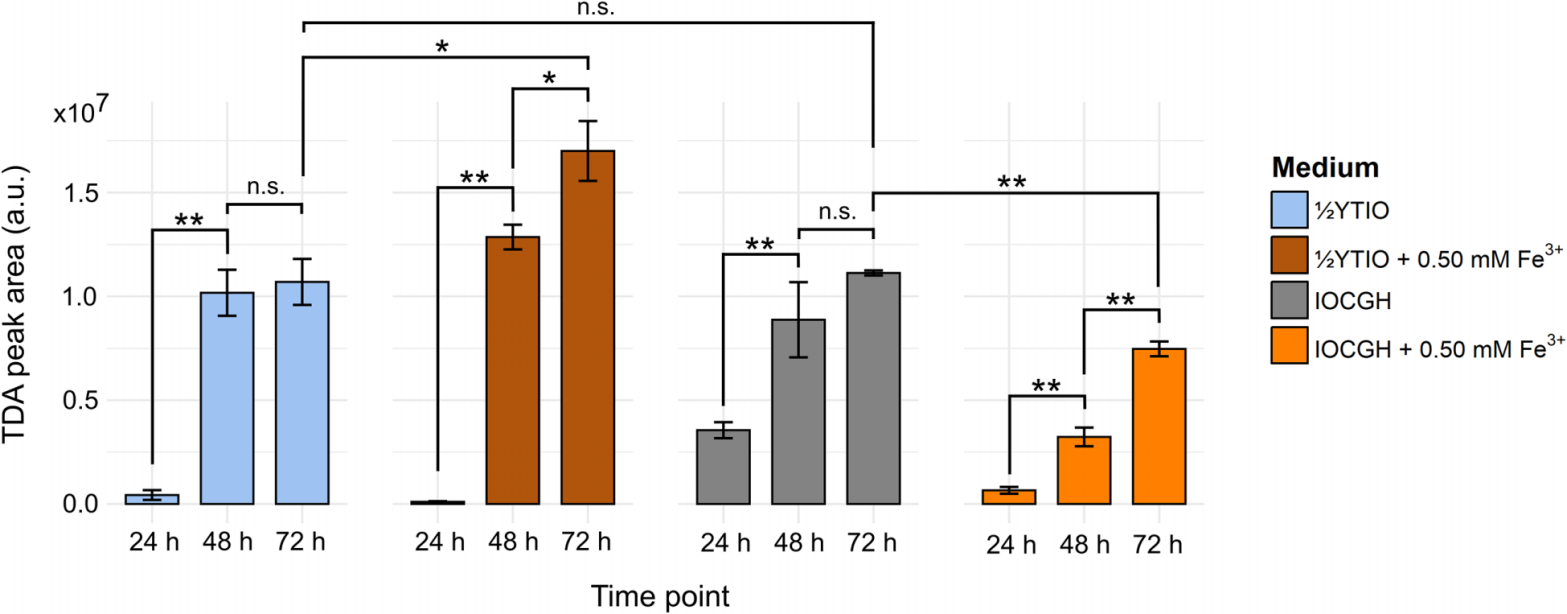
TDA production depends on media composition, presence of iron, and time of incubation. The abundance of total TDA in cultures of *P. piscinae* S26 grown in either $\frac{1}{2}$YTIO medium or IOCGH medium, with or without 0.50 mM Fe³⁺, at three different time points (24 h, 48 h, 72 h) was measured by integration of the peak area (a.u.) of extracted ion chromatograms (EICs) of the two dominant TDA adducts, [TDA+H]^+^ (*m/z* 212.9674 ± 0.002) and [TDA+Na]^+^ (*m/z* 234.9494 ± 0.002). Samples were prepared using acidic ethyl acetate, and the peak areas therefore represent TDA along with any acid-labile structural analogues that have converted into TDA during sample preparation. Mean values of biological triplicates are shown with error bars representing standard deviations of the mean. Statistical significance between selected pairs of samples was assessed using two-sided Welch’s t-tests, with *P*-values adjusted for multiple testing using the Benjamini–Hochberg false discovery rate (FDR) procedure. Significance levels are indicated as *P* < 0.05 (*), *P* < 0.01 (**), and *P* ≥ 0.05 (n.s., not significant). Full statistical analysis shown in Supplementary tables S1 and S2.

In general, TDA concentrations increased over time with significantly higher TDA levels in culture extracts at 72 h than at 24 h within each of the four media/iron combinations (Fig. 3A) (Welch’s t-test, *P* < 0.01; Table S1). In the absence of iron, no significant difference in TDA levels was observed when comparing extracts from $\frac{1}{2}$YTIO-based cultures with extracts from IOCGH-based cultures harvested at 48 h or 72 h (Welch’s t-test, *P* < 0.05, Table S2). By contrast, TDA levels in extracts from cultures with and without ferric iron differed significantly, but in opposite directions depending on the base medium. While iron supplementation to IOCGH-based cultures led to lower TDA levels at both 48 h and 72 h, consistent with the repression of transcription observed from the fluorescent reporter assays (Fig. 2D), iron-supplemented $\frac{1}{2}$YTIO-based cultures surprisingly had higher TDA levels than the corresponding iron-limited cultures at 48 h and 72 h (Fig. 3, Table S2). Ferric iron supplementation caused slight acidification of media, but while the pH of IOCGH-based cultures with and without iron remained constant throughout the experiment, the pH values of $\frac{1}{2}$YTIO-based cultures increased to around 7.5–8.0 by 48–72 h (Figure S3A), with no difference between iron-supplemented and iron-limited conditions. TDA is known to complex with ferric iron (D’Alvise et al. 2016), but no signals or isotope patterns corresponding to the iron adducts of TDA ([2 M − 2H + Fe]⁺ and [3 M − 2H + Fe]⁺) were detected in the culture extracts by MS above noise levels. Culture extracts were also analysed by UHPLC-UV/Vis, with absorbance at 385 nm revealing a chromatographic peak corresponding to TDA and related chromophores (D’Alvise et al. 2016) with similar relative quantities between samples with and without iron (Figure S3B) as those quantities measured by our MS-based detection.

### Antimicrobial activity of $\frac{1}{2}$YTIO-based cultures decreases with cultivation time

Antimicrobial activity, as measured by well-diffusion assays, has routinely been used as a proxy for the level of TDA in bacterial cultures (Bruhn et al. 2005, Berger et al. 2011, Raina et al. 2016). Hence, sterile-filtered supernatants were prepared from the *P. piscinae* S26 WT cultures mentioned in the previous section and tested for their ability to inhibit the growth of an agar-embedded population of the TDA-susceptible pathogen *V. anguillarum* strain 90–11–286 (Pedersen et al. 1997).

In contrast to results from similar well-diffusion assays conducted previously (D’Alvise et al. 2016), we observed that supernatants from iron-limited cultures demonstrated visible inhibition zones (Table 4). Nevertheless, supernatants from iron-supplemented cultures showed larger inhibition zones than their iron-limited counterparts. Samples from 24 h cultures were an exception, which could be because the production of TDA happens at a relatively late time point in non-shaken cultures (Fig. 3). Generally, the inhibition zones obtained with supernatants from iron-supplemented cultures collected at 48 h and 72 h were comparable in size regardless of base medium (IOCGH or $\frac{1}{2}$YTIO). The most conspicuous difference between the two media is that iron-limited $\frac{1}{2}$YTIO-based culture supernatants only gave visible inhibition zones at 24 h and 48 h, but not at 72 h unless acidified. Generally, acidification of culture supernatants consistently enhanced antimicrobial activity, as expected from literature (Tsubotani et al. 1984, D’Alvise et al. 2016). By comparison, non-acidified iron-limited IOCGH-based culture supernatants gave inhibition zones at all time points, with highest activity at 72 h. The surprising loss of detectable antimicrobial activity in $\frac{1}{2}$YTIO-based cultures at 72 h was observed in all biological replicates of every repetition of the experiment (four times in total). In one repetition, the iron chelation activity was tested in parallel (Figure S4), revealing that the drop in antimicrobial activity below the limit of detection at 72 h did not correlate with loss of iron chelation activity. On the contrary, iron chelation activity increased with cultivation time.

**Table 4:** Inhibitory activity of *P. piscinae* S26 culture supernatants.

Media	[Fe^3+^] (mM)	Treatment	Inhibition zone diameter (mm)		
			24 h	48 h	72 h
$\frac{1}{2}$ YTIO	0.00	Non-acidified	9.7 ± 1.2**	16.0 ± 0.0	< LOD
		Acidified	13.3 ± 1.5	25.3 ± 0.6	24.0 ± 0.0
	0.50	Non-acidified	8.3 ± 1.2**	21.3 ± 0.6	18.3 ± 0.6
		Acidified	12.0 ± 0.0*	26.0 ± 1.0	25.0 ± 0.0
IOCGH	0.00	Non-acidified	15.0 ± 0.0	16.7 ± 0.6	17.7 ± 0.6
		Acidified	23.0 ± 0.0	24.3 ± 0.6	24.0 ± 0.0
	0.50	Non-acidified	13.7 ± 0.6	18.0 ± 0.0	20.0 ± 1.0
		Acidified	18.7 ± 0.6	22.3 ± 0.6	23.0 ± 0.0

* = unclear inhibition zone, ** = foggy inhibition zone., LOD = limit of detection (7 mm)

Sterile-filtered supernatants were made from non-shaken cultures of *P. piscinae* S26 (in biological triplicates) in different media and at three time points. The ability of acidified and non-acidified supernatants to inhibit growth of *Vibrio anguillarum* 90–11–286 is measured based on the diameter of the inhibition zone (including the diameter of the well).

### Acidification affects the abundance of TDA structural analogues

**Figure 4:**
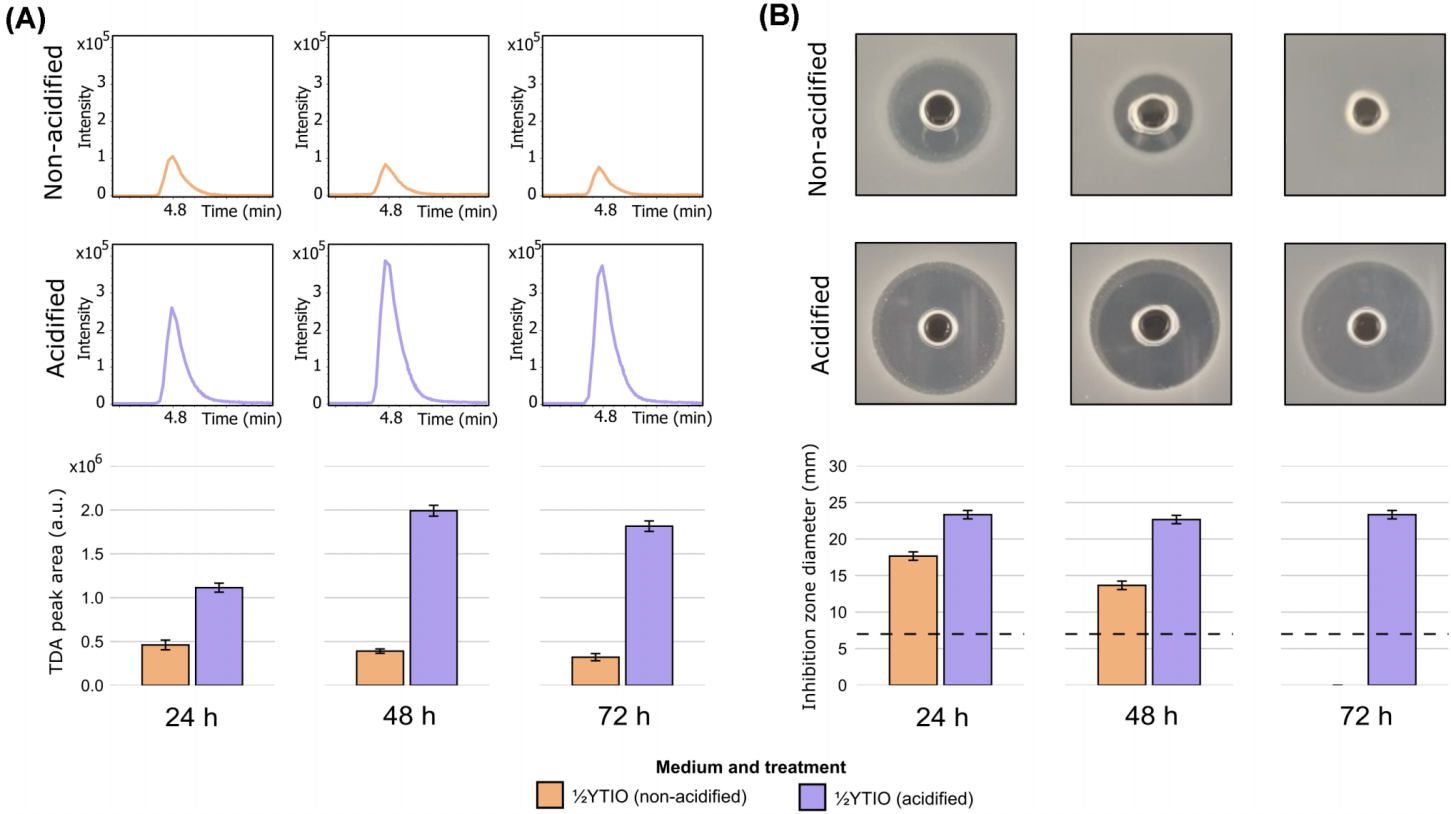
Acidification enhances tropodithietic acid (TDA) levels and antimicrobial activity of supernatants from $\frac{1}{2}$YTIO-based cultures of *P. piscinae* S26. Sterile-filtered culture supernatants from *P. piscinae* S26 grown in $\frac{1}{2}$YTIO medium for 24 h, 48 h, or 72 h were either acidified with hydrochloric acid (purple) or left non-acidified (orange) prior to analysis. (A) Abundance of tropodithietic acid (TDA) in culture supernatants was quantified by UHPLC–MS. Representative extracted ion chromatograms (EICs) of the deprotonated molecular ion of TDA ([TDA–H]⁻, *m/z* 210.9528 ± 0.002) are shown for each condition and time point. Mean integrated TDA peak areas (a.u. ± SD) from biological triplicates are summarized in the bar charts below. (B) Antimicrobial activity of culture supernatants was assessed using agar well-diffusion assays against *Vibrio anguillarum* 90–11–286. Representative inhibition zones are shown (same scale for all images), with corresponding mean inhibition zone diameters in millimeters (mm ± SD) plotted below. The dashed line indicates the limit of detection (7 mm).

The lack of detectable antimicrobial activity in sterile-filtered supernatants from $\frac{1}{2}$YTIO-based cultures at 72 h unless acidified encouraged us to compare metabolites present in the acidified and non-acidified supernatants, focusing on TDA and its structural analogues, crucially without using acidic solvents during sample preparation. After multiple attempts of using solid-phase extraction and direct injection, we found freeze-drying and methanol redissolution as the most robust protocol, followed by UHPLC-MS/MS detection. While the precursor ion of TDA was chemically detected in the non-acidified supernatants at all time points (Fig. 4A), the concentration had decreased by 72 h. By comparison, acidification of supernatants to pH 1.5–2.0 led to increased TDA concentration at every time point. These chemical differences were paralleled by the antimicrobial activity of these samples (Fig. 4B), in which acidified supernatants produced larger inhibition zones than the non-acidified equivalents. The increase in antimicrobial activity following acidification therefore reflects a true increase in the pool of TDA available for detection and bioactivity.

**Figure 5:**
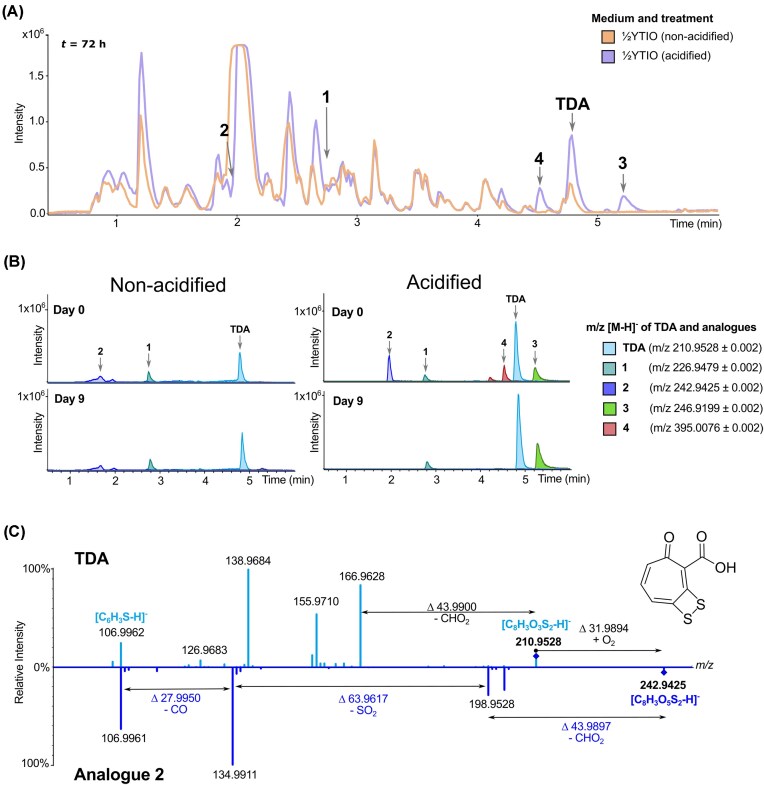
Acidification affects the stability of tropodithietic acid structural analogs. (A) Stacked Base Peak Chromatograms (BPC) acquired in ESI negative mode of chemical extracts of acidified (purple) and non-acidified (orange) sterile-filtered supernatants of 72 h cultures of *P. piscinae* S26 WT. Tropodithietic acid (TDA) elutes at 4.8 min. Numbers (1–4) highlights several detected analogues of TDA, all sharing the characteristic MS/MS fragment [C_6_H_3_S-H]^−^ (*m/z* 106.9961 ± 0.002). (B) Comparative Extracted Ion Chromatograms (EICs) of TDA and its four potential analogues in representative non-acidified and acidified samples injected on the same day as the sample preparation (Day 0) and 9 days after sample preparation (Day 9). (C) MS/MS spectrum of TDA compared in mirror to the annotated ones of the new potential structural analogue **2**.

The culture supernatant metabolome did undergo changes upon acidification, appreciated by comparing base-peak chromatograms of acidified and non-acidified supernatants (Fig. 5A). Apart from the TDA peak area increasing, two other peaks at nearby retention times can be detected in the acidified supernatants. UHPLC-MS/MS analysis and accurate mass detection revealed four potential structural analogues (**1–4**) of TDA (Table S3), recognized by their shared fragment ion (*m/z* 106.9962 ± 0.002, [C_6_H_3_S-H]^−^) characteristic of the tropolone backbone. One of the analogues was putatively identified as hydroxytropodithietic acid (compound **1**, *m*/*z* 226.9479 ± 0.002, [M-H]^−^), which has been reported previously (Liang 2003), while compounds **2–4** represent hitherto undescribed molecules (Table S3). Notably, some of these analogues were either absent or markedly reduced in the acidified samples unless the extracts were injected and analyzed immediately after sample preparation (Fig. 5B, Table S3). In particular, compound **2** (*m/z* 242.9425 ± 0.002, [M-H]^−^), hypothesized to be dihydroxytropodithietic acid (with two additional oxygen atoms relative to TDA, Fig. 5C), was readily detected and seemingly stable in non-acidified extracts but became undetectable in the acidified extract following 9 days of storage (up to 24 h storage at 4°C, followed by 8-day storage at −20°C). Similarly, compound **4** (*m/z* 395.0076 ± 0.002, [M-H]^−^) was only observed in acidified samples immediately after preparation and disappeared when samples were reanalyzed (Table S3). The exact structural identity of these acid-labile analogues remains to be determined. In contrast, the TDA peak itself remained stable under the same conditions. These observations indicate that certain TDA precursors or derivatives are unstable under acidification condition.

## Discussion

Our study reveals a previously unrecognized aspect of TDA biosynthesis: the core TDA biosynthetic genes are regulated by iron in a manner that closely mirrors biosynthetic systems of canonical siderophores (Andrews and Robinson 2003, McRose et al. 2018) and aligns with the ability of TDA to chelate iron. This finding places TDA within a broader iron-responsive metabolic framework, a regulatory context that has not been shown before at a transcriptional level. In growth medium with glucose and casamino acids, such as IOCGH, the transcriptional regulatory response to iron directly correlated with chemical detection of TDA, demonstrating that iron availability controls both gene expression and metabolite production in low-complexity medium. However, consistent with observations by D’Alvise et al. (2016), this relationship diverges in complex media like $\frac{1}{2}$YTIO, where total TDA-related products accumulate predominantly under iron-supplemented conditions despite higher transcriptional activity under iron-limited conditions. This discrepancy could, in principle, arise from analytical biases such as matrix effects or differences in physicochemical conditions between treatments. However, matrix effects were unlikely to account for the observed trends, as relative differences in TDA abundance detected by UHPLC-MS were reproduced by UHPLC-UV/Vis analysis targeting the characteristic absorbance at 385 nm. Likewise, pH differences did not explain the increased TDA levels in iron-supplemented $\frac{1}{2}$YTIO cultures, as both iron-limited and iron-replete conditions converged to similar pH values (7.5–8.0) at later time points. Instead, this decoupling of transcription from total metabolite output by the TDA biosynthetic pathway in $\frac{1}{2}$YTIO-based cultures with and without iron suggests that additional factors, such as post-transcriptional controls, precursor availability, or environmental constraints, modulates TDA production in complex growth medium.

In contrast to previous results from a related *Phaeobacter* sp. (D’Alvise et al. 2016), we observed antimicrobial activity of supernatants derived from iron-limited cultures of *P. piscinae* S26 in both media used in this study. However, as $\frac{1}{2}$YTIO-based cultures reached 72 h of growth, antimicrobial activity declined despite continued expression of the TDA biosynthesis genes, revealing a previously undescribed temporal decoupling between TDA biosynthetic pathway activity and antimicrobial output. Antimicrobial activity increased to detectable levels upon acidification of the culture supernatant, paralleled by an increase in TDA relative abundance (peak area). Conversely, UHPLC-MS/MS analyses further highlighted several TDA structural analogues in the non-acidified, non-antimicrobial culture supernatant, some of which were no longer detected following acidification. Apart from hydroxytropodithietic acid identified previously (Liang 2003), the chemical identity of these structural analogues remains unknown. The chemical mechanism responsible for their hypothesized conversion into TDA (e.g. protonation of heteroatoms or hydrolysis) can also not be unambiguously established based on the present data. However, the observed acid-dependent disappearance of TDA analogues and concomitant increase in the main TDA signal is consistent with a pH-driven interconversion between structurally related species. These observations highlight the capacity of the TDA biosynthetic pathway to generate a chemically diverse pool of related metabolites rather than a single end product. Indeed, other secondary metabolites such as methyl troposulfenin (Phippen et al. 2019) and roseobacticides (Wang et al. 2016) also depend on an intact TDA biosynthetic gene cluster. It is suggested that the structural analogues of TDA contribute to the observed dynamics of bioactivity in complex cultures. For instance, methyl troposulfenin, reported as a TDA analogue with reduced antimicrobial activity (Phippen et al. 2019), may function as a less bioactive reservoir. Consistent with this interpretation, while antimicrobial activity waned over time in the $\frac{1}{2}$YTIO-based cultures, iron-chelating activity persisted in non-acidified supernatants, implying that certain TDA structural analogues retain siderophore-like properties while exhibiting diminished or no antimicrobial function.

Marine bacterial siderophores provide a diverse context for interpreting these findings (Butler 2005, Vraspir and Butler 2009, Boiteau et al. 2016). Beyond canonical siderophores, many marine bacteria produce non-canonical, multifunctional secondary metabolites that combine antimicrobial activity, iron binding, signaling, or detoxification properties (Miethke and Marahiel 2007, Hider and Kong 2010, Xie and Wu 2014). For instance, sideromycins such as albomycin and ferrimycin combine antibiotic and siderophore functionalities, exploiting iron uptake systems to deliver cytotoxic agents to competing bacteria (Sackmann et al. 1962, Lin et al. 2018, Butler 2022). Similarly, compounds such as pyoverdines represent evolutionary adaptations where antimicrobial or photoreactive properties coexist with iron-binding functions (Meyer 2000).

Structurally, TDA belongs to the tropolone class of compounds, characterized by a non-benzenoid aromatic ring system with known metal-chelating properties (Duan et al. 2020). While tropolones can form detectable complexes with iron, their role as siderophores remains to be unraveled. A relevant parallel exists in *Burkholderia plantarii* (formerly *Pseudomonas plantarii*), which produces iron-binding tropolone derivatives that primarily act as antimicrobials, with iron chelation likely serving as a secondary function (Azegami et al. 1988). It is therefore plausible that the iron-binding capacity of TDA represents an exaptation originally evolved for antimicrobial activity but later co-opted to assist in iron homeostasis. Likewise, the moderate ferric iron affinity places TDA among weak siderophores (D’Alvise et al. 2016, Henriksen et al. 2022), which is consistent with the liquid CAS assay results shown here. This moderate reaction to iron parallels the regulation of 7-hydroxytropolone in *Pseudomonas donghuensis*, where the compound is produced in larger amounts than the high iron-affinity siderophore pyoverdine, but its biosynthesis is not strictly controlled by iron repression (Jiang et al. 2016). In *P. donghuensis*, 7-hydroxytropolone acts both as an iron-scavenger and an antibiotic (Meck et al. 2014, Jiang et al. 2016), with lower affinity for ferric iron but the advantage of simpler, less energetically-costly biosynthesis (Ravel and Cornelis 2003, Cornelis 2010) as compared to pyoverdine. A similar pattern may apply to TDA in *P. piscinae* S26; its relatively weak ferric iron affinity compared to canonical siderophores may be compensated for by its abundant production, which is not tightly regulated by iron limitation, and thus serving as a low-cost, rapid-response iron scavenger, especially in the harsh marine environments.

TDA-producing bacteria, particularly members of the *Roseobacter* group, are widely distributed across diverse marine habitats, including algal microbiomes and marine biofilms (Sonnenschein et al. 2017b, Bentzon-Tilia et al. 2025). The moderate affinity of TDA for ferric iron likely reflects an evolutionary compromise between securing iron and maintaining symbiotic balance with algal hosts. In mutualistic associations, excessive iron scavenging by microbes can induce host stress or even disrupt community structure (Seyedsayamdost et al. 2011, 2014). In this context, the intermediate iron-binding strength of TDA and its structural analogues may help *P. piscinae* acquire sufficient iron without depriving the host or destabilizing the microbiome (Sonnenschein et al. 2017b). Moreover, *P. piscinae* S26 does not appear to produce other siderophores than those arising from an intact and active TDA biosynthetic pathway, lacking, for example, the genetic potential for the siderophore *roseobactin* produced by the related species *P. inhibens* during its transition from mutualist to parasite with its algal host (Wang et al. 2022). Relying solely on TDA-like siderophores may reflect niche adaptations to the algal phycosphere, where localized gradients of iron, pH, and organic matter favor flexible, low-cost strategies. *Phaeobacter* spp. commonly inhabit such host-associated environments, where localized chemistry can differ markedly from bulk seawater. During algal senescence, released metabolites such as aromatic acids may generate transient acidic micro-niches (Seyedsayamdost et al. 2011, Cirri and Pohnert 2019, Wang et al. 2022), consistent with the acid-dependent shifts in TDA composition observed here. At the same time, these bacteria also occur in environments with stable, near-neutral pH (Sonnenschein et al. 2021, Svendsen et al. 2024, Bentzon-Tilia et al. 2025), indicating that pH-driven interconversion of TDA analogues is likely context-dependent, relevant in some niches while less influential in more stable or less complex marine conditions.

Collectively, our results support a role for TDA biosynthesis in iron homeostasis. While its siderophore activity is lower than that of canonical siderophores, the environmentally responsive regulation, chemical diversity, and biological versatility of TDA and its structural analogues may offer adaptive benefits in marine host-associated environments. TDA belongs to the class of non-canonical siderophores that do not fit the traditional definition but nonetheless may play critical roles in microbial survival, competition, and symbiosis. Future research should aim to elucidate the molecular mechanisms of TDA-iron complex transport and uptake, explore the ecological functions and transformation of TDA structural analogues, and address the hypothesized TDA-mediated iron-scavenging within algal microbiomes. Understanding such multifunctional secondary metabolites will deepen our knowledge of microbial chemical ecology and provide new perspectives on how bacteria adapt to complex and competitive environments like the marine phycosphere *via* adjusting the production of secondary metabolites.

## Supplementary Material

fiag080_Supplemental_File
