# Supplementary material for: Re-examining the iron paradox: the influence of growth medium on regulation and functionality of the tropodithietic acid (TDA) biosynthetic pathway in Phaeobacter piscinae
Source: FEMS Microbiol Ecol. 2026 Jul 17;102(8):fiag080. doi: 10.1093/femsec/fiag080 (PMC13421778; doi:10.1093/femsec/fiag080)
Supplement: fiag080_Supplemental_File [file fiag080_supplemental_file.docx]

**Supplementary data**

**Re-examining the iron paradox: the influence of growth medium on regulation and functionality of the tropodithietic acid (TDA) biosynthetic pathway in *Phaeobacter piscinae***

**Lauge Alfastsen**^1^**, Morgane Mauduit**^1^**, Michael Scott Cowled**^1,2^**, Nikoletta Angela Vig**^1^**, Robert Friis-Møller**^1^**, Frederik Valdemar Holck Reimert**^1^**, Morten Dencker Schostag**^1,3^**, Aaron John Christian Andersen**^1^**, Lone Gram**^1^**, and Sheng-Da Zhang**^1^*****

^1^ Center for Microbial Secondary Metabolites (CeMiSt), Department of Biotechnology and Biomedicine, Technical University of Denmark, DK-2800 Kgs Lyngby, Denmark

^2^ Current address: Department of Biochemistry and Pharmacology, Bio21 Institute, The University of Melbourne, Parkville, VIC 3010, Australia

^3^ Current address: Novonesis, DK-2880 Bagsværd, Denmark

Keywords (6 words): tropodithietic acid, iron, GFP-reporter fusion, biosynthesis, antimicrobial activity, chemical diversity

**Supplementary Table S1. Statistical comparison of mean TDA concentrations in chemical extracts within each medium.** Pairwise comparisons were performed independently within each of the four media groups using two-sided Welch’s t-tests. Group means represent three independent biological replicates (n = 3); technical replicates were averaged prior to statistical testing. *t* denotes the t-statistic and *df* the Welch-adjusted degrees of freedom. *P*-FDR indicates p-values adjusted for multiple testing using the Benjamini–Hochberg false discovery rate procedure. Data corresponds to results shown in Figure 3.

| **Media** | **Time point** **1** | **Time point  2** | **Mean_ time point 1** | **Mean_ time point 2** | ***t*** | ***df*** | **p_FDR** |
| --- | --- | --- | --- | --- | --- | --- | --- |
|  |  |  |  |  |  |  |  |
| ½YTIO | 24 h | 48 h | 430097.7 | 10172425.7 | -14.88 | 2.18 | 0.00467 |
| ½YTIO | 48 h | 72 h | 10172425.7 | 10695956.3 | -0.58 | 4.00 | 0.59394 |
| ½YTIO | 24 h | 72 h | 430097.7 | 10695956.3 | -15.70 | 2.18 | 0.00467 |
| ½YTIO + Fe^3+^ | 24 h | 48 h | 104462.7 | 12857247.1 | -37.08 | 2.01 | 0.00212 |
| ½YTIO + Fe^3+^ | 48 h | 72 h | 12857247.1 | 17003241.3 | -4.60 | 2.66 | 0.02488 |
| ½YTIO + Fe^3+^ | 24 h | 72 h | 104462.7 | 17003241.3 | -20.30 | 2.00 | 0.00361 |
| IOCGH | 24 h | 48 h | 3554660.8 | 8873474.0 | -4.97 | 2.18 | 0.04782 |
| IOCGH | 48 h | 72 h | 8873474.0 | 11127129.4 | -2.15 | 2.02 | 0.16339 |
| IOCGH | 24 h | 72 h | 3554660.8 | 11127129.4 | -32.39 | 2.40 | 0.00094 |
| IOCGH + Fe^3+^ | 24 h | 48 h | 656648.8 | 3229970.9 | -9.32 | 2.52 | 0.00515 |
| IOCGH + Fe^3+^ | 48 h | 72 h | 3229970.9 | 7469677.2 | -12.80 | 3.80 | 0.00043 |
| IOCGH + Fe^3+^ | 24 h | 72 h | 656648.8 | 7469677.2 | -30.13 | 2.80 | 0.00039 |

**Supplementary Table S2. Statistical comparison of mean TDA concentrations in chemical extracts at each time point.** Pairwise comparisons were performed independently at each time point using two-sided Welch’s t-tests. Group means represent three independent biological replicates (n = 3); technical replicates were averaged prior to statistical testing. *t* denotes the t-statistic and *df* the Welch-adjusted degrees of freedom. *P*-FDR indicates p-values adjusted for multiple testing using the Benjamini–Hochberg false discovery rate procedure. Data corresponds to results shown in Figure 3.

| **Time point** | **Group 1** |  | **Group 2** | **Mean_ group1** | **Mean_ group2** | ***t*** | ***df*** | **p_FDR** |
| --- | --- | --- | --- | --- | --- | --- | --- | --- |
|  |  |  |  |  |  |  |  |  |
| 24 h | ½YTIO |  | IOCGH | 430097.7 | 3554660.8 | -11.97 | 3.31 | 0.00465 |
| 24 h | ½YTIO |  | ½YTIO + Fe^3+^ | 430097.7 | 104462.7 | 2.38 | 2.06 | 0.16421 |
| 24 h | IOCGH |  | IOCGH + Fe^3+^ | 3554660.8 | 656648.8 | 11.98 | 2.69 | 0.00618 |
| 24 h | ½YTIO + Fe^3+^ |  | IOCGH + Fe^3+^ | 104462.7 | 656648.8 | -5.77 | 2.13 | 0.03716 |
| 48 h | ½YTIO |  | IOCGH | 10172425.7 | 8873474.0 | 1.06 | 3.31 | 0.36051 |
| 48 h | ½YTIO |  | ½YTIO + Fe^3+^ | 10172425.7 | 12857247.1 | -3.69 | 3.06 | 0.04984 |
| 48 h | IOCGH |  | IOCGH + Fe^3+^ | 8873474.0 | 3229970.9 | 5.24 | 2.25 | 0.04984 |
| 48 h | ½YTIO + Fe^3+^ |  | IOCGH + Fe^3+^ | 12857247.1 | 3229970.9 | 22.36 | 3.72 | 0.00025 |
| 72 h | ½YTIO |  | IOCGH | 10695956.3 | 11127129.4 | -0.67 | 2.05 | 0.57031 |
| 72 h | ½YTIO |  | ½YTIO + Fe^3+^ | 10695956.3 | 17003241.3 | -6.01 | 3.75 | 0.01049 |
| 72 h | IOCGH |  | IOCGH + Fe^3+^ | 11127129.4 | 7469677.2 | 16.82 | 2.47 | 0.00787 |
| 72 h | ½YTIO + Fe^3+^ |  | IOCGH + Fe^3+^ | 17003241.3 | 7469677.2 | 11.12 | 2.24 | 0.01049 |

**Supplementary Table S3.** **LC-MS/MS profiling of TDA and its structural analogs found in sterile-filtered supernatant from ½YTIO-based cultures after 72h of growth.** Compounds were detected in negative ionization mode [ESI(–)] and are listed with observed mass-to-charge ratios (*m/z*), retention time (*t_R_*), molecular formulas, and mass error (ppm). Tentative compound identities were assigned based on spectral matching and database references if the compound has been described elsewhere. Presence/absence was recorded in acidified and non-acidified samples at two time points: immediately after sample preparation (Day 0) and after <24 hours of storage at 4°C and 8 days of storage at -20°C (Day 9).

| **ID** | **ESI(-) MS observed *m/z*** | ***t_R_* (min)** | **Molecular formula** | **Err. (ppm)** | **Identity  (tentative*)** | **Ref.** | **Present in  non-acidified samples** | | | **Present in acidified samples** | | | |  |
| --- | --- | --- | --- | --- | --- | --- | --- | --- | --- | --- | --- | --- | --- | --- |
|  |  |  |  |  |  |  | **Day 0** | **Day 9** | | **Day 0** | | **Day 9** | |  |
| **TDA** | 210.9528 | 4.79 | C_8_H_4_O_3_S_2_ | -0.1 | Tropodithietic acid | Liang, 2001 | Yes | | Yes | | Yes | | Yes | |
| **1** | 226.9479 | 2.78 | C_8_H_4_O_4_S_2_ | -0.7 | Hydroxy tropodithietic acid* | Liang, 2001 | Yes | | Yes | | Yes | | Yes | |
| **2** | 242.9425 | 1.95 | C_8_H_4_O_5_S_2_ | 1.2 | Unknown | N/A | Yes | | Yes | | Yes | | No | |
| **3** | 246.9199 | 5.25 | C_7_H_4_O_4_S_3_ | -0.2 | Unknown | N/A | Yes | | Yes | | Yes | | Yes | |
| **4** | 395.0076 | 4.53 | C_20_H_12_O_5_S_2_ | -5.7 | Unknown | N/A | No | | No | | Yes | | No | |


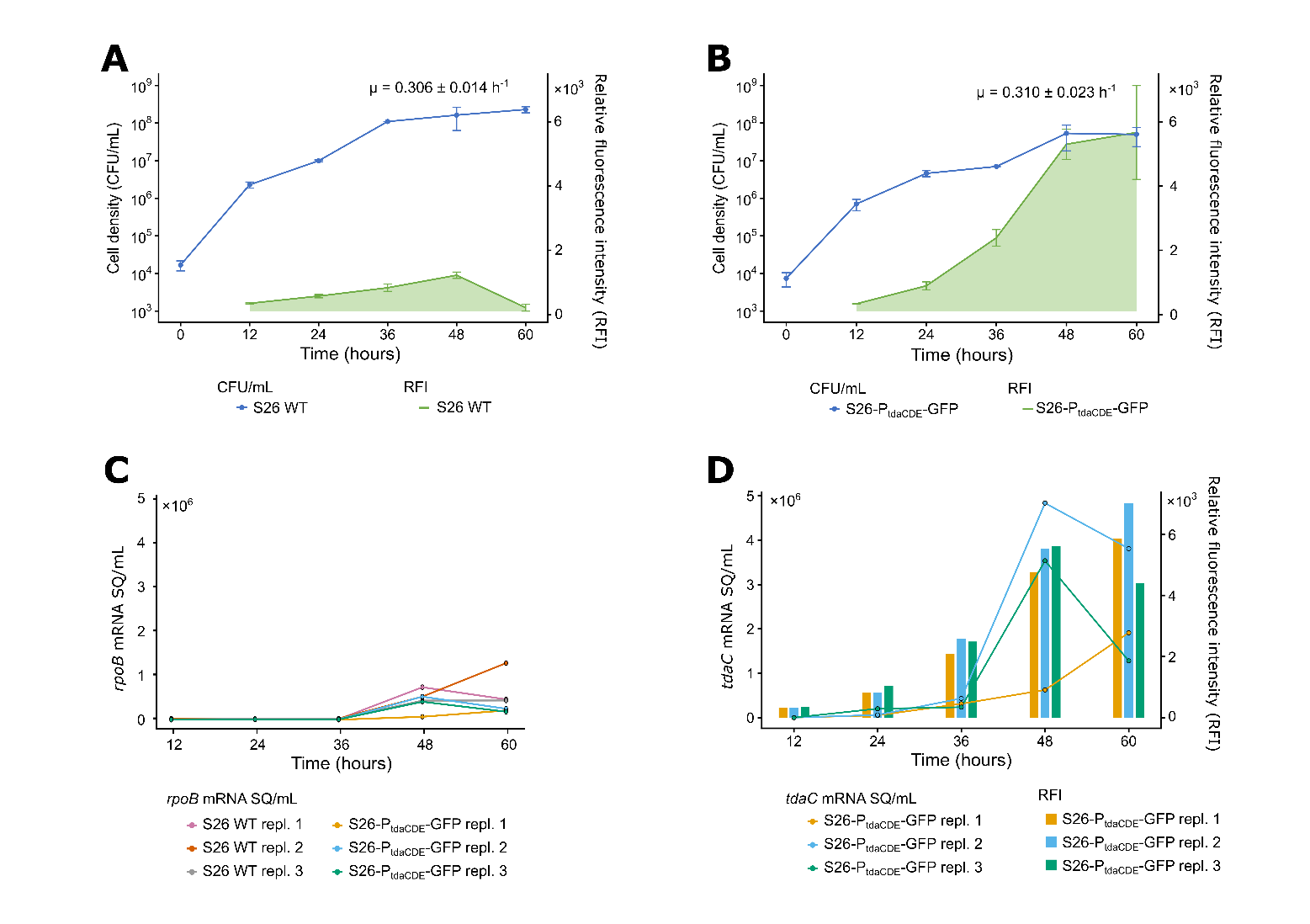


**Supplementary Figure S1. Transcription of *rpoB* and *tdaC* in individual biological replicates and correspondence with GFP reporter output.** Cultures of wild-type *Phaeobacter piscinae* S26 and the reporter strain *P. piscinae* S26-P*_tdaCDE_*-GFP were grown non-shaken in IOCGH medium in Erlenmeyer flasks for 60 hours, with growth (CFU/mL), green fluorescence (Relative Fluorescence Intensity) and starting quantities of *rpoB* and *tdaC* mRNA measured every 12 hours. (**A**) S26 WT showed typical growth dynamics and only low, stable background fluorescence. (**B**) The S26-P*_tdaCDE_*-GFP reporter strain displayed similar growth kinetics to S26 WT (no significant difference between calculated growth rates, Welch’s t-test, p = 0.80) but exhibited a strong increase in fluorescence from 24–48 h, consistent with promoter activation during the transition to stationary phase. Calculated growth rates (μ) are shown in the panels as mean ± standard error from three biological replicates. **(C)** Starting quantities (SQ/mL) of *rpoB* mRNA measured by RT-qPCR in three biological replicates of wild-type *Phaeobacter piscinae* S26 WT and three biological replicates of S26-P*_tdaCDE_*-GFP. mRNA levels were similar across replicates up to 36 h, with divergence appearing at 48–60 h, particularly in S26 WT repl. 2. **(D)** Comparison of *tdaC* transcript abundance (curves; SQ/mL) and GFP fluorescence (bars; RFI) in the three replicates of the reporter strain. The *tdaC* expression and GFP fluorescence showed strong concordance across time points (Pearson r = 0.84), demonstrating that the fluorescence of the S26-P*_tdaCDE_*-GFP reporter strain closely reflects endogenous *tdaC* transcription dynamics.


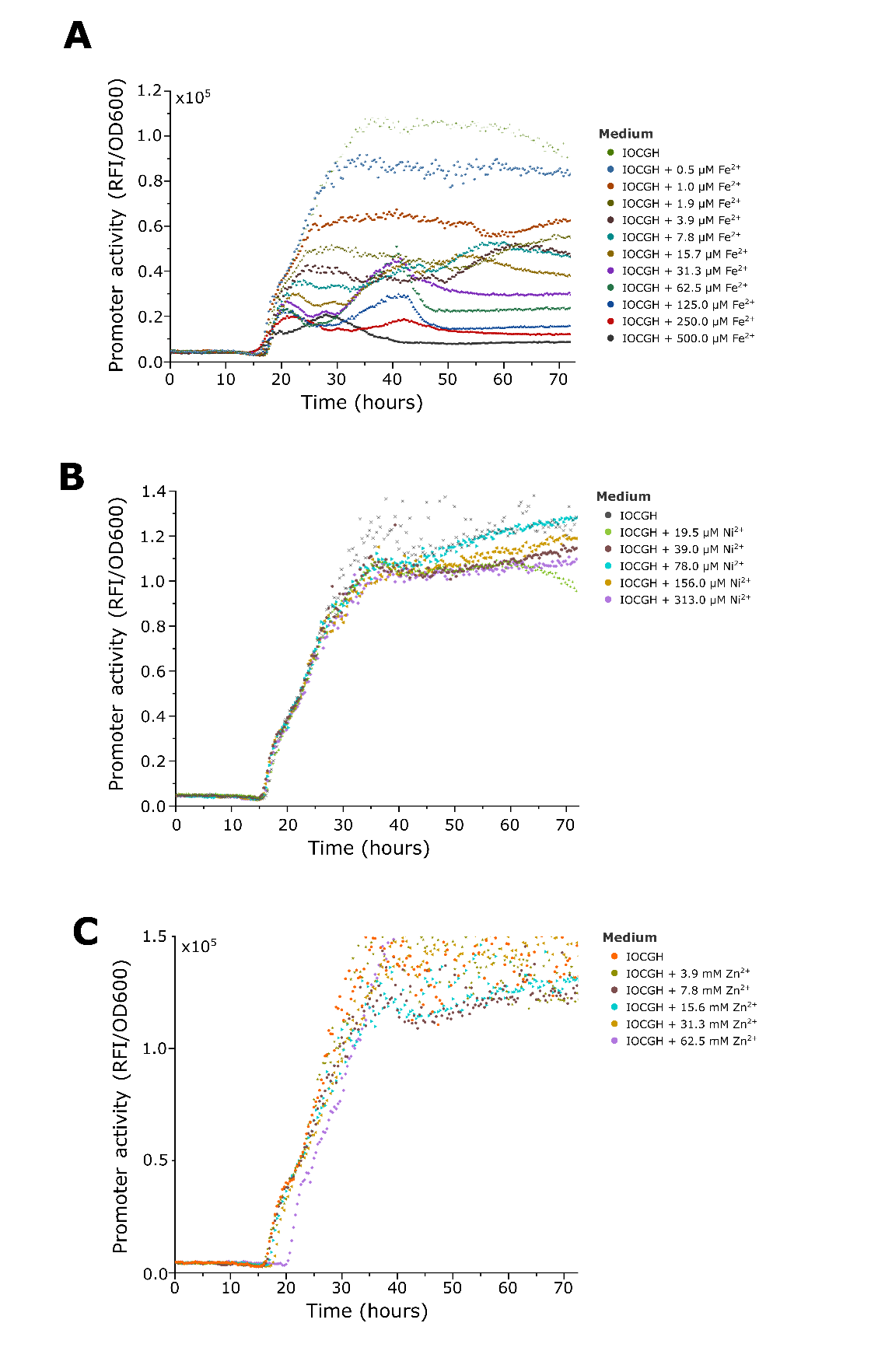


**Supplementary Figure S2. The transcriptional activity of the *tdaCDE* promoter in IOCGH medium supplemented with ferrous iron, nickel or zinc.** The reporter strain *Phaeobacter piscinae* S26-P*_tdaCDE_*-GFP was grown in IOCGH medium containing (**A**) ferrous iron (Fe^2+^), (**B**) nickel (Ni**^2+^**), or (**C**) zinc (Zn^2+^) in a range of indicated concentrations. The *tdaCDE* promoter activity was quantified as GFP relative fluorescence intensity (RFI) normalized to optical density (RFI/OD₆₀₀). Supplementation with ferrous iron caused repression of *tdaCDE* promoter activity, whereas addition of nickel or zinc did not significantly affect the activity, demonstrating iron-specific regulation of the TDA biosynthetic pathway.

**
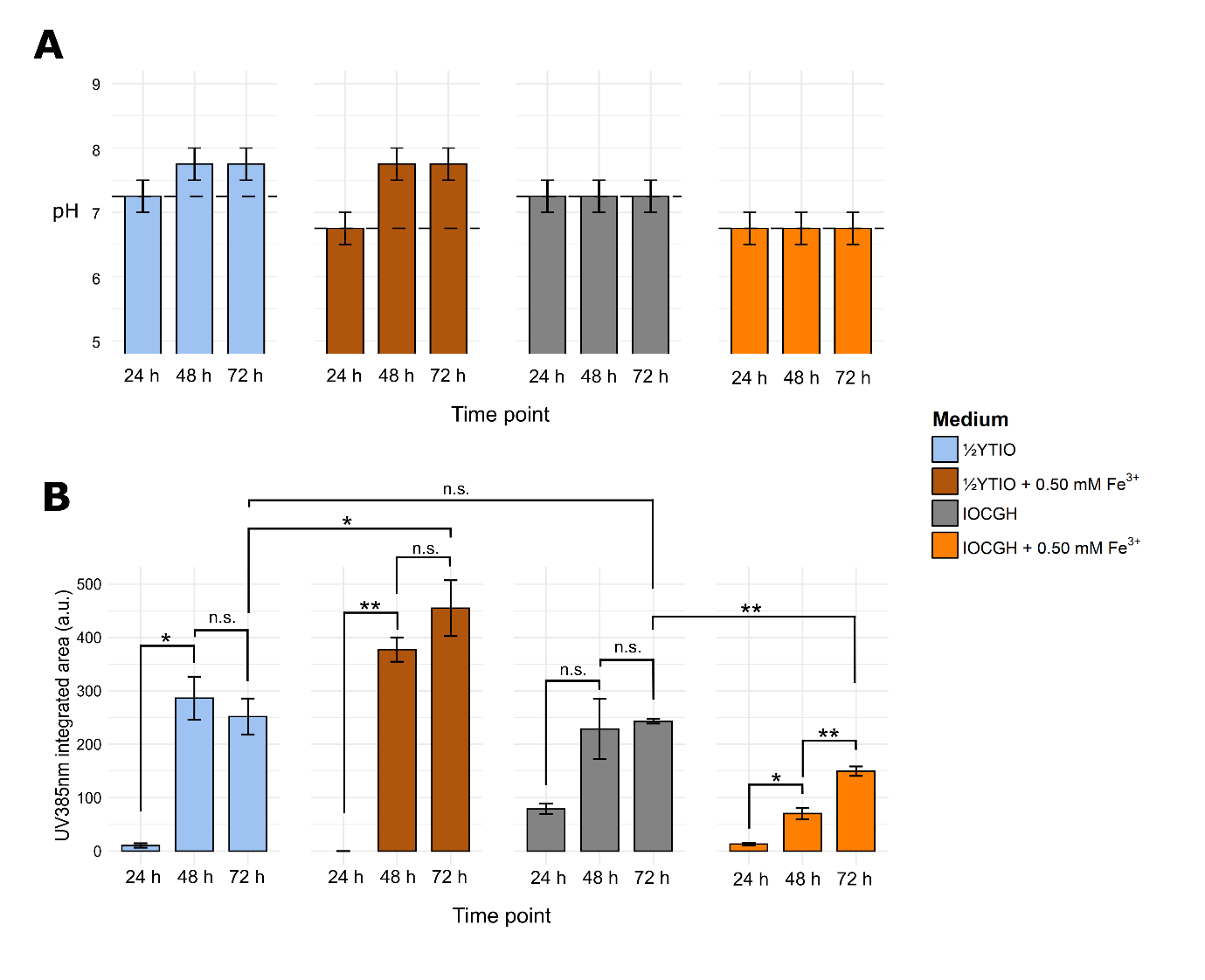
**

**Supplementary figure S3. pH measurements of culture aliquots and UV/Vis absorption at 385 nm of culture extracts.** (**A**) The pH of *Phaeobacter piscinae* S26 cultures from different media and harvested at different time points was measured using pH indicator strips from Cytiva (pH 4.5-10, Cat # 2614-991). Dashed lines indicate pH of the corresponding non-inoculated media control. Error bars indicate the level of uncertainty (±0.25) of the pH indicator. (**B**) Ultraviolet (UV)-visible absorbance signals at 385 nm were recorded from culture extracts following UHPLC separation, expressed as integrated peak areas (a.u.), reflecting the relative abundance of TDA and structurally related compounds. Bars represent mean values of biological triplicates, and error bars indicate standard deviations. Statistical significance between selected time points was assessed using two-sided Welch’s t-tests with Benjamini–Hochberg correction; significance levels are indicated as *p* < 0.05 (*), *p* < 0.01 (**), and not significant (n.s.). Culture extracts described here are the same culture extracts presented in Figure 3.


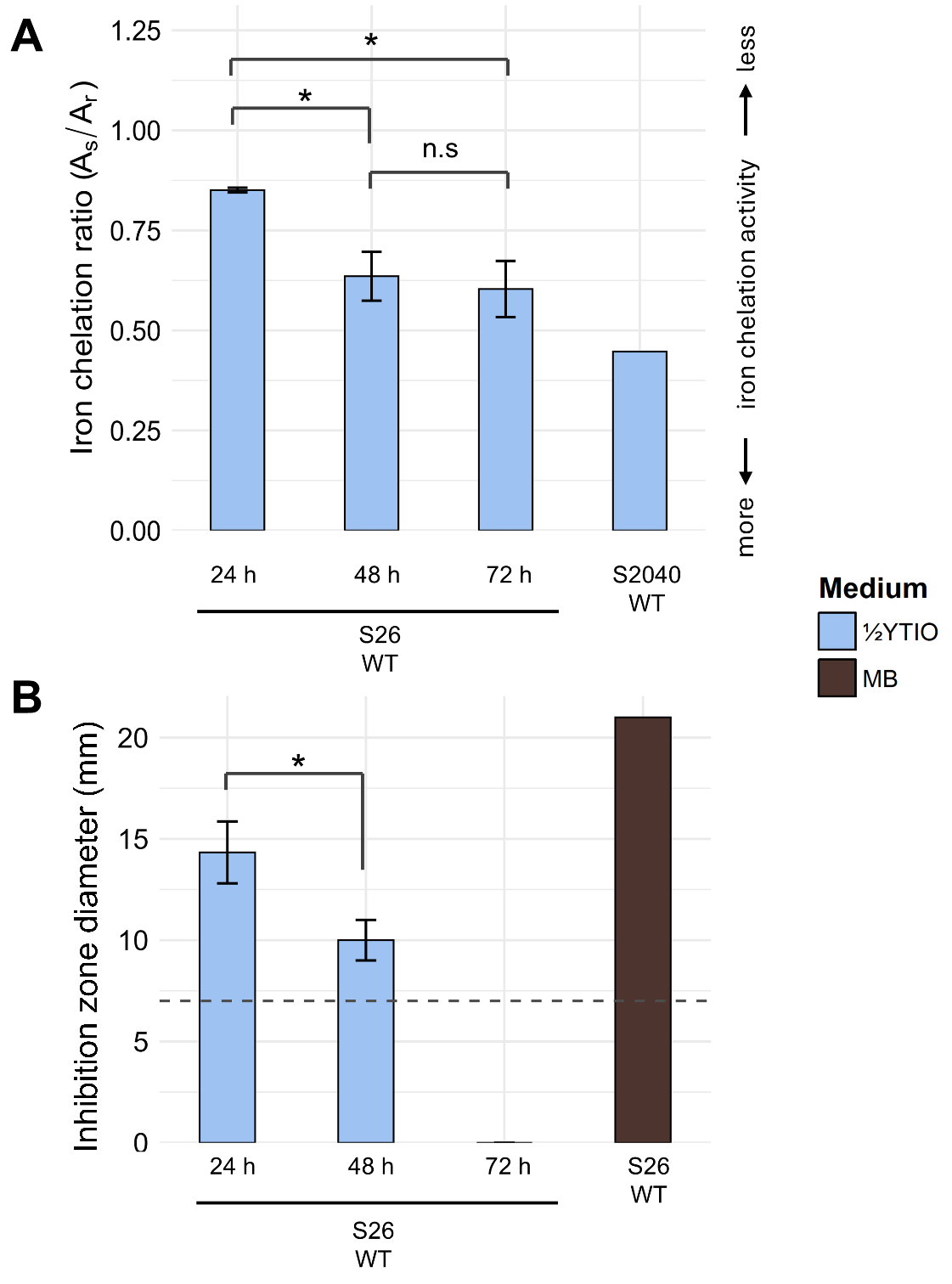


**Supplementary Figure S4. Antimicrobial activity and iron-chelating activity are not coupled.** (**A**) Iron-chelating activity of sterile-filtered culture supernatants from *Phaeobacter piscinae* S26 WT grown in ½YTIO medium for 24–72 h, measured using the liquid CAS assay and expressed as the ratio of absorbance at 630 nm (Aₛ/Aᵣ), where lower values indicate higher iron-chelating activity. (**B**) Antimicrobial activity of corresponding culture supernatants from panel A assessed by agar diffusion assay against *Vibrio* *anguillarum* 90-11-286, shown as inhibition zone diameters (mm). Mean values of biological triplicates are shown with error bars representing standard deviations. Statistical significance was assessed using two-sided Welch’s *t*-tests; p < 0.05 (*).
